# ﻿Morphological and phylogenetic analyses reveal two new species of *Tubeufia* (Tubeufiales, Tubeufiaceae) from freshwater habitats in China

**DOI:** 10.3897/mycokeys.121.158724

**Published:** 2025-08-21

**Authors:** Xiao-Yan Ma, Fu Tian, Jiang-Fen Feng, Min-Min Wang, Huan-Huan Shi, Jian Ma

**Affiliations:** 1 School of Food and Pharmaceutical Engineering, Guizhou Institute of Technology, Guiyang 550003, China School of Food and Pharmaceutical Engineering, Guizhou Institute of Technology Guiyang China; 2 Guizhou Key Laboratory of Agricultural Microbiology, Guizhou Academy of Agricultural Sciences, Guiyang 550009, China Guizhou Key Laboratory of Agricultural Microbiology, Guizhou Academy of Agricultural Sciences Guiyang China; 3 Guizhou Industry Polytechnic College, Guiyang 550008, China Guizhou Industry Polytechnic College Guiyang China

**Keywords:** 2 new species, helicosporous hyphomycetes, phylogeny, saprobic fungi, taxonomy

## Abstract

*Tubeufia* is a genus of helicosporous hyphomycetes distinguished by a high degree of morphological variation in its asexual morphs. During an investigation of helicosporous fungi in tropical regions, four fungal strains were isolated from submerged decaying wood in Hainan Province, southern China. Multigene phylogenetic analyses based on a combined ITS–LSU–*tef1-α–rpb2* sequence dataset were conducted to determine the phylogenetic placement of the four fungal strains, which showed two distinct new species. Morphological characteristics were also used to support their taxonomic delimitation in addition to the molecular phylogeny. Two novel species, *Tubeufia yanuodaensis* and *T. yinggelingensis*, are described and illustrated.

## ﻿Introduction

*Tubeufia* was described by Penzig and Saccardo (1897) with *T. javanica* as the type species based on morphological characteristics. Currently, *Tubeufia* comprises 86 species, of which 27 are found in freshwater habitats, 44 in terrestrial habitats, and 15 in both freshwater and terrestrial habitats (Penzig and Saccardo 1897; Barr 1979; Rossman 1987; Lu et al. 2017, 2018a, 2018b, 2022, 2023; Lu and Kang 2020; Tian et al. 2022; Ma et al. 2024; Lu et al. 2025). These species are primarily saprobic on decaying wood in tropical and temperate regions—predominantly in China and Thailand (Chaiwan et al. 2017; Dai et al. 2017; Doilom et al. 2017; Lu et al. 2017, 2018a, 2018b, 2022, 2023; Luo et al. 2017; Kuo and Goh 2018a, 2018b; Tibpromma et al. 2018; Lu and Kang 2020; Li et al. 2022; Tian et al. 2022; Ma et al. 2023, 2024; Lu et al. 2025).

*Tubeufia* species are widely distributed in Austria, Bermuda, Brazil, Canada, China, Colombia, Cuba, India, New Zealand, Panama, Peru, South Africa, Sri Lanka, Tanzania, Thailand, Trinidad, Uganda, Venezuela, and the USA (Morgan 1892; Talbot 1956; Moore 1957; Rao and Rao 1964; Deighton and Pirozynski 1966; Munk 1966; Ellis 1971; Panwar et al. 1973; Barr 1979, 1980; Barr and Rogerson 1983; Goos 1985, 1990; Matsushima 1987, 1993; Rossman 1987; Holubová-Jechová 1988; Ma et al. 2023, 2024; Lu et al. 2025). The asexual morph of *Tubeufia* is considered the most morphologically diverse among helicosporous hyphomycetes (Ma et al. 2023, 2024). For instance, the conidial morphology of *Tubeufia* species exhibits a wide range of forms, including dorsiventrally curved, coiled, ovate, ellipsoid to ovoid, spherical to obclavate, and subreniform conidia, occasionally bearing one or more small, globose secondary conidia (Tsui and Berbee 2006; Tsui 2007; Zhao et al. 2007; Lu et al. 2018b; Ma et al. 2023, 2024).

In this study, two saprobic *Tubeufia* species were collected from submerged decaying wood in Hainan Province, China. Based on morphological characteristics and multigene phylogenetic analyses, two novel species, *Tubeufia yanuodaensis* and *T. yinggelingensis*, are described and illustrated. Comparisons with closely related taxa are also provided to support their taxonomic placements.

## ﻿Materials and methods

### ﻿Sample collection, specimen examination, and isolation

Samples of submerged decaying wood pieces were collected from Hainan Province in southern China between October 2021 and July 2022, and the collection details were recorded (Rathnayaka et al. 2024). The wood samples were taken to the mycology laboratory at Guizhou Institute of Technology for examination. Fresh specimens were incubated in zip-lock bags and sterile, moist plastic boxes at room temperature for two weeks. The microscopic features were examined and photographed using a stereomicroscope (SMZ-168, Nikon, Japan) and an ECLIPSE Ni compound microscope (Nikon, Tokyo, Japan) with a Canon 90D digital camera.

Single spore isolations were performed on PDA plates following the methods described by Senanayake et al. (2020), and the germinated helicosporous conidia were aseptically transferred to fresh PDA plates. Mycelium was grown on PDA and incubated at 25 °C for 30–39 days—the morphological characteristics, such as color, shape, and size, were recorded. Dried fungal specimens were deposited in the Herbarium of Kunming Institute of Botany, Chinese Academy of Sciences (Herb. HKAS), Kunming, China, and the Herbarium of Guizhou Academy of Agriculture Sciences (Herb. GZAAS), Guiyang, China. Pure cultures were deposited in the Guizhou Culture Collection, China (GZCC), Guiyang, China. The MycoBank numbers of newly obtained species were registered in the MycoBank database (https://www.mycobank.org/).

### ﻿DNA extraction, PCR amplification, and sequencing

Fresh fungal mycelia were scraped from colonies grown on PDA plates and transferred to a 1.5 mL microcentrifuge tube using a sterilized lancet for genomic DNA extraction. Genomic DNA was extracted using the Biospin Fungus Genomic DNA Extraction Kit (BioFlux, China). ITS5/ITS4, LR0R/LR5, fRPB2-5F/fRPB2-7cR, and EF1-983F/EF1-2218R were employed to amplify the internal transcribed spacer (ITS; White et al. 1990), large ribosomal subunit (LSU; Vilgalys and Hester 1990), RNA polymerase II second largest subunit (*rpb2*; Liu et al. 1999), and translation elongation factor 1-α (*tef1-α*; Rehner and Buckley 2005) sequence fragments, respectively. DNA preparation was conducted in a 25 μL mixture, which included 1 μL DNA, 1 μL of the forward and reverse primers each, and 22 μL of 1.1× T3 Supper PCR Mix (Qingke Biotech, Chongqing, China). The conditions for the polymerase chain reaction (PCR) correspond to those reported by Ma et al. (2023). The PCR products were purified and sequenced with the same primers at Beijing Tsingke Biotechnology Co., Ltd.

### ﻿Phylogenetic analyses

The forward and reverse sequence data of our new taxa were checked and assembled using BioEdit v.7.0.5.3 (Hall 1999) and SeqMan v.7.0.0 (DNASTAR, Madison, WI, USA; Swindell and Plasterer 1997), respectively. The sequences incorporated in this study were downloaded from GenBank (Table 1; https://www.ncbi.nlm.nih.gov/). Multiple sequences were aligned using MAFFT v.7.473 (https://mafft.cbrc.jp/alignment/server/; Katoh and Standley 2013; Katoh et al. 2019). The dataset was trimmed using trimAl v.1.2rev59 software (Capella-Gutiérrez et al. 2009). A combined sequence dataset was created using SequenceMatrix-Windows-1.7.8 software (Vaidya et al. 2011). The Maximum Likelihood (ML) tree was constructed using the IQ-TREE web server (http://iqtree.cibiv.univie.ac.at/; Nguyen et al. 2015). Bayesian Inference (BI) analysis was conducted following the methodology described by Ma et al. (2022).

**Table 1.** Taxa used in this study, along with their corresponding GenBank accession numbers for the DNA sequences.

Taxon	Strain	GenBank Accessions			
		ITS	LSU	tef1-α	rpb2
*Acanthohelicospora aurea*	GZCC 16-0060	KY321323	KY321326	KY792600	MF589911
*Acanthohelicospora guianensis*	UAMH 1699	AY916479	AY856891	-	-
*Neohelicomyces xiayadongensis*	MUCL 15702	AY916459	AY856873	-	-
*Tubeufia abundata*	MFLUCC 17-2024	MH558769	MH558894	MH550961	MH551095
*Tubeufia acropleurogena*	CGMCC 3.25582^T^	PP626645	PP639501	PP596394	PP596513
*Tubeufia africana*	BCRC FU30906	LC371247	LC424099	-	LC494221
*Tubeufia africana*	BCRC FU30840	LC371248	LC424100	-	LC494223
*Tubeufia africana*	BCRC FU30867	LC371251	LC424103	-	LC494222
*Tubeufia aquatica*	MFLUCC 16-1249^T^	KY320522	KY320539	KY320556	MH551142
*Tubeufia aquatica*	MFLUCC 17-1794	MH558770	MH558895	MH550962	MH551096
*Tubeufia bambusicola*	MFLUCC 17-1803^T^	MH558771	MH558896	MH550963	MH551097
*Tubeufia baomeilingensis*	CGMCC 3.25580^T^	PP626648	PP639504	PP596397	PP596515
*Tubeufia baoshanensis*	ZHKUCC 23-0682^T^	PQ608348	PQ608180	PQ628189	PV367523
*Tubeufia brevis*	MFLUCC 17-1799^T^	MH558772	MH558897	MH550964	MH551098
*Tubeufia brunnea*	MFLUCC 17-2022^T^	MH558773	MH558898	MH550965	MH551099
*Tubeufia chiangmaiensis*	MFLUCC 17-1801	MH558774	MH558899	MH550966	MH551100
*Tubeufia chiangmaiensis*	MFLUCC 11-0514^T^	KF301530	KF301538	KF301557	-
*Tubeufia chiangraiensis*	MFLUCC 25-0189^T^	PQ608350	PQ608182	PQ628191	PV367525
*Tubeufia chlamydospora*	MFLUCC 16-0223^T^	MH558775	MH558900	MH550967	MH551101
*Tubeufia cocois*	MFLUCC 22-0001^T^	OM102541	OL985957	OM355486	OM355491
*Tubeufia coffeae*	MFLUCC 25-0190^T^	PQ608353	PQ608185	PQ628194	PV367528
*Tubeufia cylindrothecia*	MFLUCC 16-1283	KY320518	KY320535	KY320552	MH551143
*Tubeufia cylindrothecia*	MFLUCC 17-1792	MH558776	MH558901	MH550968	MH551102
*Tubeufia denticulata*	CGMCC 3.25583^T^	PP626653	PP639509	PP596402	PP596520
*Tubeufia dictyospora*	MFLUCC 17-1805^T^	MH558778	MH558903	MH550970	MH551104
*Tubeufia dictyospora*	MFLUCC 16-0220	MH558777	MH558902	MH550969	MH551103
*Tubeufia eccentrica*	GZCC 16-0084	MH558781	MH558906	MH550973	MH551107
*Tubeufia eccentrica*	MFLUCC 17-1524^T^	MH558782	MH558907	MH550974	MH551108
*Tubeufia entadae*	MFLU 18-2102^T^	MK347727	MK347943	-	-
*Tubeufia fangchengensis*	MFLUCC 17-0047^T^	MH558783	MH558908	MH550975	MH551109
*Tubeufia filiformis*	MFLUCC 16-1128^T^	-	KY092407	KY117028	MF535284
*Tubeufia filiformis*	MFLUCC 16-1135	KY092416	KY092411	KY117032	MF535285
*Tubeufia formosiformis*	BCRC FU30850	LC371249	LC424101	-	LC494219
*Tubeufia formosiformis*	BCRC FU30757^T^	LC193730	LC201751	-	LC494220
*Tubeufia formosiformis*	BCRC FU30851^T^	LC371250	LC424102	-	LC494218
*Tubeufia freycinetiae*	MFLUCC 16-0252^T^	MH275089	MH260323	MH412786	-
*Tubeufia geniculata*	BCRC FU30849^T^	LC335817	-	-	-
*Tubeufia geniculata*	NCYU-U2-1B	LC335816	-	-	-
*Tubeufia guangxiensis*	MFLUCC 17-0045^T^	MG012025	MG012018	MG012004	MG012011
*Tubeufia guangxiensis*	MFLUCC 17-0046	MH558784	MH558909	MH550977	MH551111
*Tubeufia guttulata*	GZCC 23-0404^T^	OR030841	OR030834	OR046678	OR046684
*Tubeufia hainanensis*	GZCC 22-2015^T^	OR030842	OR030835	OR046679	OR046685
*Tubeufia hechiensis*	MFLUCC 17-0052^T^	MH558785	MH558910	MH550978	MH551112
*Tubeufia hyalospora*	MFLUCC 15-1250^T^	MH558786	MH558911	MH550979	-
*Tubeufia inaequalis*	MFLUCC 17-0053^T^	MH558789	MH558914	MH550982	MH551115
*Tubeufia inaequalis*	MFLUCC 17-1989	MH558790	MH558915	MH550983	MH551116
*Tubeufia javanica*	MFLUCC 12-0545^T^	KJ880034	KJ880036	KJ880037	-
*Tubeufia krabiensis*	MFLUCC 16-0228^T^	MH558792	MH558917	MH550985	MH551118
*Tubeufia latispora*	MFLUCC 16-0027^T^	KY092417	KY092412	KY117033	MH551119
*Tubeufia laxispora*	MFLUCC 16-0219	KY092414	KY092409	KY117030	MF535286
*Tubeufia laxispora*	MFLUCC 16-0232^T^	KY092413	KY092408	KY117029	MF535287
*Tubeufia lilliputea*	NBRC 32664	AY916483	AY856899	-	-
*Tubeufia liyui*	GZCC 22-2030^T^	OP888466	OP888465	OP856589	OP856588
*Tubeufia longihelicospora*	MFLUCC:21-0151	OL606156	OL606149	OL964520	OL964526
*Tubeufia longihelicospora*	MFLUCC 16-0753^T^	MZ538531	MZ538565	MZ567106	-
*Tubeufia longiseta*	MFLUCC 15-0188^T^	KU940133	-	-	-
*Tubeufia machaerinae*	MFLUCC 17-0055	MH558795	MH558920	MH550988	MH551122
*Tubeufia mackenziei*	MFLUCC 16-0222^T^	KY092415	KY092410	KY117031	MF535288
*Tubeufia muriformis*	GZCC 22-2039^T^	OR030843	OR030836	OR046680	OR046686
*Tubeufia nigroseptum*	CGMCC 3.20430^T^	MZ092716	MZ853187	OM022002	OM022001
*Tubeufia pandanicola*	MFLUCC 16-0321^T^	MH275091	MH260325	-	-
*Tubeufia parvispora*	MFLUCC 17-2009	MH558798	MH558923	MH550991	MH551125
*Tubeufia parvispora*	MFLUCC 16-0324^T^	MH275090	MH260324	MH412787	MH412761
*Tubeufia roseohelicospora*	MFLUCC 17-1797	MH558800	MH558925	MH550993	MH551127
*Tubeufia roseohelicospora*	MFLUCC 15-1247^T^	KX454177	KX454178	-	MH551144
*Tubeufia rubra*	GZCC 16-0083	MH558802	MH558927	MH550995	MH551129
*Tubeufia rubra*	GZCC 16-0081^T^	MH558801	MH558926	MH550994	MH551128
*Tubeufia sahyadriensis*	NFCCI 4252 RAJ/99.1^T^	MH033849	MH033850	MH033851	-
*Tubeufia sahyadriensis*	NFCCI RAJ 99.2	MN393081	MN393082	MN393083	-
*Tubeufia sessilis*	MFLUCC 16-0021^T^	MH558803	-	MH550996	MH551130
*Tubeufia subrenispora*	CGMCC 3.25560^T^	PP781938	PP781939	PP785815	PP785813
*Tubeufia sympodihylospora*	GZCC 16-0051	MH558805	MH558929	MH550998	MH551132
*Tubeufia sympodihylospora*	MFLUCC 17-0044^T^	MH558806	MH558930	MH550999	MH551133
*Tubeufia sympodihylospora*	GZCC 16-0049	MH558804	MH558928	MH550997	MH551131
*Tubeufia sympodilaxispora*	GZCC 16-0058	MH558807	MH558931	MH551000	MH551134
*Tubeufia sympodilaxispora*	MFLUCC 17-0048^T^	MH558808	MH558932	MH551001	MH551135
*Tubeufia taiwanensis*	BCRC FU30844^T^	LC316605	-	-	-
*Tubeufia tectonae*	MFLUCC 17-1985	MH558810	MH558934	MH551003	MH551137
*Tubeufia tectonae*	MFLUCC 12-0392^T^	KU144923	KU764706	KU872763	-
*Tubeufia tratensis*	MFLUCC 17-1993^T^	MH558811	MH558935	MH551004	MH551138
*Tubeufia tropica*	GZCC 23-0219^T^	PP626675	PP639531	-	PP596536
*Tubeufia xylophila*	MFLUCC 17-1520	MH558813	MH558937	MH551006	MH551140
*Tubeufia xylophila*	GZCC 16-0038	MH558812	MH558936	MH551005	MH551139
Tubeufia yanuodaensis	GZCC 23-0488^T^	PQ098484	PQ098521	PV768323	PV768332
Tubeufia yanuodaensis	GZCC 24-0165	PV730408	PV730412	PV768324	PV768333
Tubeufia yinggelingensis	GZCC 23-0525^T^	PQ098483	PQ098520	PV768321	PV768330
Tubeufia yinggelingensis	GZCC 24-0166	PV730407	PV730411	PV768322	PV768331
Tubeufiaceae sp.	BCC 3512	AY916484	AY856905	-	-

Note: T denotes ex-type strain. Newly generated sequences are indicated in bold black. “-” means no data available in GenBank.

Phylogenetic trees were visualized using FigTree v.1.4.4 and edited with Adobe Illustrator CC 2019 (v.23.1.0; Adobe Systems, USA). Photo plates were assembled using Adobe Photoshop CC 2019 (Adobe Systems, USA). Measurements were made using Tarosoft (R) Image Frame Work software.

### ﻿Phylogenetic analysis results

**Figure 1.**
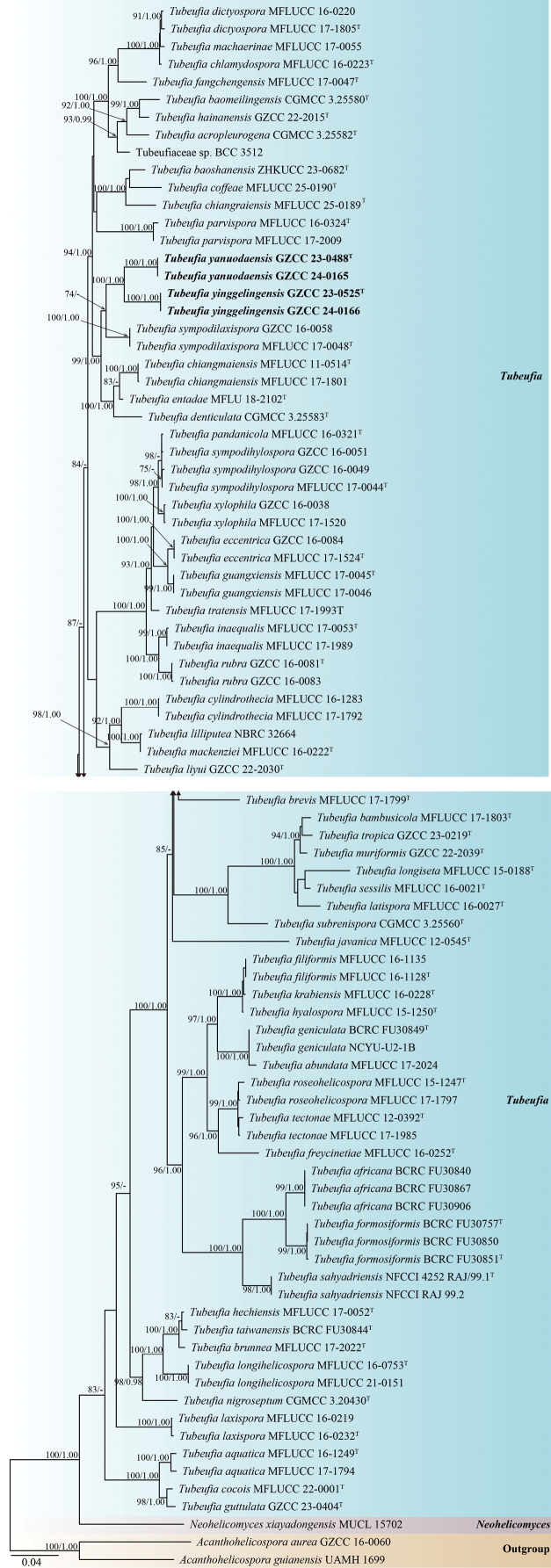
Phylogenetic tree generated from the ML analysis based on a combined dataset of LSU, ITS, *tef1-α*, and *rpb2* sequence data. Bootstrap support values from ML analyses ≥ 75% and Bayesian posterior probabilities (BYPP) ≥ 0.95 are indicated at the nodes as ML/BYPP, respectively. The ML and BI analyses yielded similar tree topologies. The tree is rooted with *Acanthohelicospora aurea*GZCC 16-0060 and *A. guianensis* UAMH 1699. The newly obtained strains are indicated in bold black. Ex-type strains are denoted with “^T^”.

The phylogenetic positions of the newly isolated taxa were inferred based on partial nucleotide sequences of the LSU, ITS, *tef1-α*, and *rpb2* gene regions. A total of 88 strains, including our newly isolated strains and two outgroups, were analyzed. The concatenated sequence matrix consisted of 3,339 characters (LSU = 844 bp, ITS = 538 bp, *tef1-α* = 912 bp, and *rpb2* = 1,045 bp). The best-scoring ML tree generated is shown in Fig. 1, with a final log-likelihood value of –23,679.213.

The multigene phylogenetic tree (Fig. 1) shows that the four new collections represent two distinct species within *Tubeufia*. The four newly obtained isolates, GZCC 23-0488 and GZCC 24-0165, form a sister lineage to GZCC 23-0525 and GZCC 24-01656 with 100% ML/1.00 BYPP support.

## ﻿Taxonomy

### ﻿ Tubeufia yanuodaensis

Taxon classificationFungiTubeufialesTubeufiaceae

X.Y. Ma, J. Ma & Y.Z. Lu sp. nov.

BB630F0D-393C-535F-8DE2-D5D4A3B49F81

904047

Fig. 2

#### Etymology.

“*yanuodaensis*” refers to the type locality, “Yanuoda Rainforest Cultural Tourist District”.

#### Holotype.

HKAS 128924.

#### Description.

***Saprobic*** on submerged decaying wood in a freshwater habitat. ***Asexual morph*** Hyphomycetous, helicosporous. ***Colonies*** on natural substrate superficial, effuse, white to pale brown. ***Mycelium*** partly superficial, composed of hyaline to pale brown, branched, septate, smooth hyphae. ***Conidiophores*** 64–83 × 6–7 μm (x̄ = 71.5 × 6.5 μm, n = 20), macronematous, mononematous, erect, cylindrical, straight or slightly flexuous, branched, septate, subhyaline to pale brown, thick-walled. ***Conidiogenous cells*** 13.5–16 × 3–5 μm (x̄ = 14.5 × 4 μm, n = 20), holoblastic, monoblastic, integrated, subcylindrical, terminal, truncate at apex after conidial secession, subhyaline to pale brown, smooth-walled. ***Conidia*** solitary, acrogenous, helicoid, 35.5–40 μm diam., and conidial filament 4.5–5 μm wide (x̄ = 37.5 × 4.8 μm, n = 20), 252–338 μm long (x̄ = 291 μm, n = 25), tightly coiled 3^1^/_4_–3^4^/_5_ times, becoming loosely coiled in water, multi-septate, slightly constricted at septa, guttulate, hyaline, smooth-walled. ***Sexual morph*** Undetermined.

**Figure 2.**
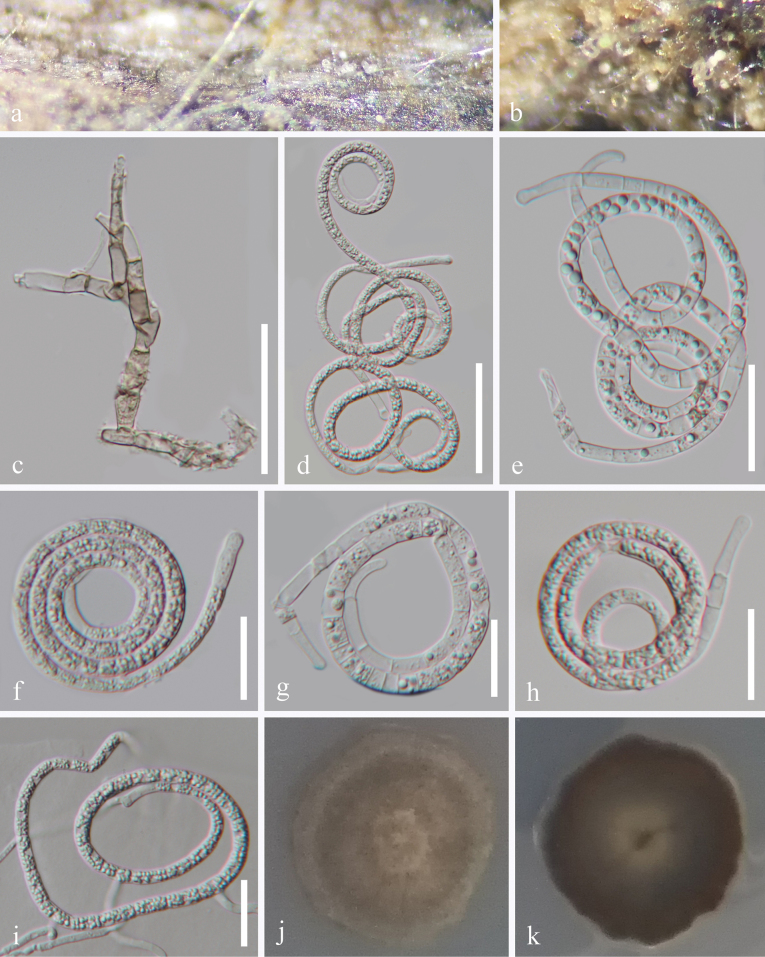
*Tubeufia yanuodaensis* (HKAS 128924, holotype). a, b. Colonies on the host surface; c. Conidiophores and conidiogenous cells; d–h. Conidia; i. Germinated conidium; j, k. Colonies on PDA, j from above, k from below. Scale bars: 40 μm (c, d); 30 μm (e); 20 μm (f–i).

#### Culture characteristics.

Conidia germinated on PDA and produced germ tubes within 9 h. Colonies on PDA are irregular with a flat surface and undulating margin, reaching 2.5 cm in diameter after 30 days at room temperature (approximately 25 °C), and are pale brown to dark brown.

#### Material examined.

China • Hainan Province, Baoting Li and Miao Autonomous County, Yanuoda Rainforest Cultural Tourist District, on submerged decaying wood in a freshwater stream, 23 October 2021, Jian Ma, Y2 (HKAS 128924, holotype), ex-type living culture GZCC 23-0488; *Ibid*., Y2-2 (GZAAS 24-0079, paratype), living culture GZCC 24-0165.

#### Notes.

In the present phylogenetic analysis, *Tubeufia yanuodaensis* (GZCC 23-0488 and GZCC 24-0165) formed a sister lineage to *T. yinggelingensis* (GZCC 23-0525 and GZCC 24-0166) with 100% ML and 1.00 BYPP statistical support (Fig. 1). Based on the molecular sequence comparison, our isolate (GZCC 23-0488, ex-type) differs from *T. yinggelingensis* (GZCC 23-0525, ex-type) by 71/857 bp for ITS (8.3%, gaps 42 bp), 7/833 bp (0.8%, gap one bp) for LSU, 20/892 bp (2.2%, without gaps) for *tef*1-α, and 45/767 bp (5.9%, gaps 3 bp) for *rpb*2. Morphologically, *Tubeufia yanuodaensis* (HKAS 128924) can be distinguished from *T. yinggelingensis* (HKAS 128856) by its larger conidia (35.5–40 μm diameter and 252–338 μm long *vs.* 22–31 μm diameter and 148–170 μm long). Therefore, *Tubeufia yanuodaensis* is introduced here as a new species based on molecular evidence and morphological comparison.

### ﻿ Tubeufia yinggelingensis

Taxon classificationFungiTubeufialesTubeufiaceae

X.Y. Ma, J. Ma & Y.Z. Lu sp. nov.

9D1D990E-6475-520C-AE51-6715D6B14F64

904048

Fig. 3

#### Etymology.

“*yinggelingensis*” refers to the type locality, “Yinggeling National Nature Reserve”.

#### Holotype.

HKAS 128856.

#### Description.

***Saprobic*** on submerged decaying wood in a freshwater habitat. ***Asexual morph*** Hyphomycetous, helicosporous. ***Colonies*** on natural substrate superficial, effuse, gregarious, with crowded, glistening conidia, white. ***Mycelium*** partly superficial, composed of hyaline to pale brown, branched, septate, guttulate, smooth hyphae. ***Conidiophores*** 21.5–143 × 4.5–7 μm (x̄ = 67.5 × 5.5 μm, n = 25), macronematous, mononematous, erect, cylindrical, flexuous, branched, septate, pale brown to brown, thick-walled. ***Conidiogenous cells*** 9–15 × 4–5 μm (x̄ = 11.5 × 4.5 μm, n = 20), holoblastic, monoblastic, integrated, terminal, cylindrical, with denticles, pale brown to brown, smooth-walled. ***Conidia*** solitary, acrogenous, helicoid, rounded at apex, developing on tooth-like protrusion, 22–31 μm diam. and conidial filament 4–6 μm wide (x̄ = 27 × 5 μm, n = 20), 148–170 μm long (x̄ = 157.5 μm, n = 20), tightly coiled 2^3^/_5_–3^1^/_4_ times, not becoming loose in water, indistinctly multi-septate, guttulate, hyaline, smooth-walled. ***Sexual morph*** Undetermined.

#### Culture characteristics.

Conidia germinated on PDA and formed germ tubes after 11 hours of incubation. Colonies on PDA are irregular with a flat surface and undulating margin, reaching 3.2 cm in diameter after 39 days at room temperature (approximately 25 °C), and are brown to dark brown in color.

**Figure 3.**
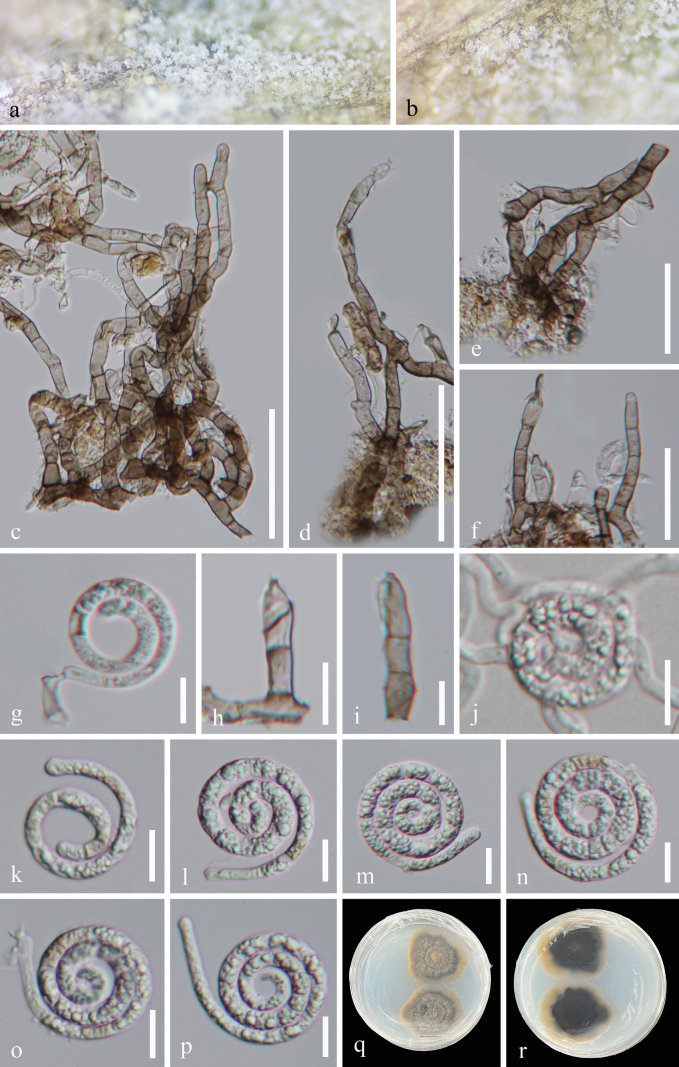
*Tubeufia yinggelingensis* (HKAS 128856, holotype). a, b. Colonies on the host surface; c–f. Conidiophores and conidiogenous cells; g–i. Conidiogenous cells and conidia; k–p. Conidia; j. Germinated conidium; q, r. Colonies on PDA, q from above, r from below. Scale bars: 50 μm (c, d); 40 μm (e, f); 10 μm (g–p).

#### Material examined.

China • Hainan Province, Yinggeling National Nature Reserve, on rotting wood in a freshwater stream, 12 July 2022, Jian Ma, YG5 (HKAS 128856, holotype), ex-type living culture GZCC 23-0525; *Ibid*., YG5-2 (GZAAS 24-0080, paratype), living culture GZCC 24-0166.

#### Notes.

Morphologically, *Tubeufia yinggelingensis* (HKAS 128856) closely resembles *T. denticulata* (HKAS 131090) in having macronematous, mononematous, erect, cylindrical, branched, septate conidiophores; holoblastic, monoblastic, integrated, cylindrical conidiogenous cells; and solitary, hyaline, guttulate, helicoid conidia (Ma et al. 2024). However, *Tubeufia yinggelingensis* (HKAS 128856) differs from *T. denticulata* (HKAS 131090) by its unique conidiogenous cells (terminal *vs.* intercalary) and larger conidia (22–31 μm diam. and conidial filament 4–6 μm wide, 148–170 μm long *vs.* 19–21 μm diam. and conidial filament 2–4 μm wide, 73.5–142.5 μm long) (Ma et al. 2024). Based on molecular phylogenetic analyses, *Tubeufia yinggelingensis* (GZCC 23-0525 and GZCC 24-0166) formed a sister clade with *T. yanuodaensis* (GZCC 23-0488 and GZCC 24-0165), which is phylogenetically distinct from *T. denticulata* (CGMCC 3.25583) (Fig. 1). Thus, based on morphological comparison and multigene phylogenetic analysis, we identified GZCC 23-0525 and GZCC 24-0166 as a new species, *Tubeufia yinggelingensis*.

## ﻿Discussion

Based on DNA sequence data and/or morphological characteristics, the genus *Tubeufia* currently comprises 88 species, including our newly described species, *T. yanuodaensis* and *T. yinggelingensis* (Karandikar and Patwardhan 1992; Lu et al. 2000; Chang 2001; Ho et al. 2002; Tsui and Berbee 2006; Tsui 2007; Zhao et al. 2007; Pande 2008; Boonmee et al. 2011, 2014, 2021; Hyde et al. 2016; Singh and Singh 2016; Li et al. 2022; Tian et al. 2022; Ma et al. 2023, 2024; Lu et al. 2025). Among them, 57 species are helicosporous fungi (Ma et al. 2024; this study). Currently, molecular data are unavailable for 30 *Tubeufia* species, and the sexual and asexual morphs have been linked in only six species (Lu et al. 2017, 2018a, 2018b, 2022, 2023; Luo et al. 2017; Kuo and Goh 2018a, 2018b, 2021; Tibpromma et al. 2018; Lu and Kang 2020; Li et al. 2022; Tian et al. 2022; Ma et al. 2023, 2024; Lu et al. 2025).

The genus *Tubeufia* exhibits diverse asexual and sexual morphologies. However, morphologically similar species within this genus typically form stable and distinct clades in multi-gene phylogenetic analyses (Fig. 1) (Zhao et al. 2007; Rajeshkumar et al. 2019; Kuo and Goh 2021; Ma et al. 2023, 2024; Lu et al. 2025). For instance, *Tubeufia africana* and *T. formosiformis* possess ellipsoidal or ovoid conidia (Kuo and Goh 2021; Ma et al. 2024), whereas *T. sahyadriensis* features maize corncob-like, dictyoseptate conidia (Rajeshkumar et al. 2019). Additionally, *Tubeufia longiseta*, *T. muriformis*, *T. sessilis*, and *T. tropica* are characterized by helicoid or curved, muriform conidia, occasionally bearing a single globose secondary conidium (Zhao et al. 2007; Ma et al. 2023, 2024), while *Tubeufia subrenispora* exhibits curved to subreniform, multicelled muriform conidia (Zhao et al. 2007; Ma et al. 2024). Moreover, *Tubeufia baoshanensis*, *T. coffeae*, and *T. chiangraiensis*—which were collected from decaying branches of *Coffea* sp.—produce broadly fusiform, cylindrical, aseptate, hyaline ascospores (Lu et al. 2025).

To date, five asexual species of *Tubeufia* with distinct conidial characteristics have been reported, primarily from China and Thailand (Zhao et al. 2007; Rajeshkumar et al. 2019; Kuo and Goh 2021; Ma et al. 2023, 2024). Among them, *Tubeufia sahyadriensis*—characterized by maize corncob-like, dictyoseptate conidia—is the only species recorded from India (Rajeshkumar et al. 2019; Ma et al. 2024).

## Supplementary Material

XML Treatment for Tubeufia yanuodaensis

XML Treatment for Tubeufia yinggelingensis

## ﻿Additional information

### Use of AI

No use of AI was reported.

### Author ORCIDs

Xiao-Yan Ma https://orcid.org/0000-0001-5874-9979

Fu Tian https://orcid.org/0000-0002-5503-4350

Jiang-Fen Feng https://orcid.org/0009-0009-2467-891X

Min-Min Wang https://orcid.org/0009-0003-6106-9992

Huan-Huan Shi https://orcid.org/0009-0001-6486-1315

Jian Ma https://orcid.org/0009-0008-1291-640X

### Data availability

All of the data that support the findings of this study are available in the main text.
